# Why using ‘harmless behaviour’, ‘risk factor’ and ‘protective factor’ as terms describing the various possible consequences of bruxism is still the best option

**DOI:** 10.1111/joor.13063

**Published:** 2020-08-19

**Authors:** Frank Lobbezoo, Jari Ahlberg, Ghizlane Aarab, Daniele Manfredini

**Affiliations:** ^1^ Department of Orofacial Pain and Dysfunction Academic Centre for Dentistry Amsterdam (ACTA) University of Amsterdam and Vrije Universiteit Amsterdam Amsterdam The Netherlands; ^2^ Department of Oral and Maxillofacial Diseases University of Helsinki Helsinki Finland; ^3^ School of Dentistry Department of Biomedical Technologies University of Siena Siena Italy

Dear Editor,

In their recent Editorial, Svensson and Lavigne^1^ stressed the need to further clarify the term ‘bruxism’ for usage in everyday clinical practice as to solve the enigmatic nature of the condition. We fully concur with the authors that bruxism remains a challenge for oral healthcare professionals, especially now that we are improving our insight into bruxism that is no longer only considered the largest ‘crook’ in dentistry, with only negative health consequences (eg advanced mechanical tooth wear, musculoskeletal pain and fractures and failures of dental restorations and implants), but that is also increasingly seen as a condition with potentially beneficial health outcomes for the individual—apart from the fact that bruxism can also be a normal physiological process and can then be considered a harmless behaviour.

Svensson and Lavigne^1^ provide a couple of examples of possible positive health outcomes of bruxism, notably its purported role in improving or restoring upper airway patency in relation to obstructive sleep apnoea, and its possible moistening effect for the oral cavity by mechanically stimulating the salivary glands. The complex collection of potentially negative and positive health outcomes, as well as bruxism being ‘just’ a harmless behaviour, has lead Svensson and Lavigne^1^ to pose the question whether a single term, bruxism, can cover all those various aspects. They take the position that the continued usage of this single term will ‘*contribute to maintain confusion about the bruxism concept, for a better understanding of the clinical significance of bruxism and best treatment planning’*.^1^ As a solution, they suggest that clinicians, teachers and researches adopt the term ‘normo‐bruxism’ for bruxism that is either associated with positive health outcomes or can be considered a harmless behaviour and the term ‘patho‐bruxism’ for bruxism that is associated with negative health outcomes. Hence, they propose a dichotomy in bruxism terminology.

However, while highly appreciating the important contributions of Svensson and Lavigne to clear the fog around bruxism during the past decades, the authors of this Letter to the Editor would like to point out that these newly proposed terms cannot be implemented without any problems, neither in research nor in clinical practice. In 2018, Lobbezoo et al^2^ published a commentary, based on international consensus, in which it was described that bruxism can be considered a harmless behaviour when it causes neither harm nor good to the individual, while it could be coined as a risk or protective factor when it is associated with negative or positive health outcomes, respectively. We would like to stress that this description was formulated for good reasons: in a single individual, bruxism can be both harmful and beneficial.

Multiple examples of this are being seen in our clinics, for example, an obstructive sleep apnoea patient bruxing to improve or restore upper airway patency who consequently suffers from musculoskeletal pain and/or advanced mechanical tooth wear at the same time. But how do we apply the newly proposed terms ‘normo‐bruxism’ and patho‐bruxism’ in such cases? In our opinion, dichotomisation does not work here. There is, after all, only one ‘indivisible’ masticatory muscle activity, as per the 2013 definition of bruxism.^3^

On the other hand, it is possible that a single activity can have multiple consequences (cf. solar radiation, which is a risk factor for several skin pathologies but at the same time a protective factor for a host of physical symptoms caused by a shortage of vitamin D). Hence, using ‘harmless behaviour’, ‘risk factor’ and ‘protective factor’ as terms describing the various possible consequences of bruxism is still the best option. It is our experience as teachers that students at all levels have no difficulties grasping the meaning of those terms. On the contrary, they all realise now that managing bruxism requires finding a balance between the condition's risk side and its protective side. Since they are all academically trained, they are well capable of going through this decision‐making process. For them, bruxism is therefore not an enigma, but rather a clinical challenge!

## DISCLOSURES

The authors declare that they received no funding for this study. They have also stated explicitly that there are no conflicts of interest in connection with this article.

## AUTHOR CONTRIBUTIONS

All authors contributed substantially to the conception of this work. FL drafted the manuscript. JA, GA and DM critically revised the manuscript. All authors have approved the final version for publication and are fully accountable for all aspects of the work.

### Peer Review

The peer review history for this article is available at https://publons.com/publon/10.1111/joor.13063.
